# Supplementary material for: Engineering ZrO2–Ru interface to boost Fischer-Tropsch synthesis to olefins
Source: Nat Commun. 2024 Jun 17;15:5143. doi: 10.1038/s41467-024-49392-w (PMC11183094; doi:10.1038/s41467-024-49392-w)
Supplement: Supplementary file 1 — Supplementary Information [file 41467_2024_49392_MOESM1_ESM.pdf]

Supplementary Information

Engineering the ZrO_2-Ru interface to boost Fischer-Tropsch synthesis to olefins

Hailing Yu[1,2,#], Caiqi Wang[1,#], Xin Xin[1,2,#], Yao Wei[2,3], Shenggang Li[1,4,*],Yunlei An[1], Fanfei Sun[5], Tiejun Lin[1,*], Liangshu Zhong[1,4,*]

[1] CAS Key Laboratory of Low-Carbon Conversion Science and Engineering, Shanghai Advanced Research Institute, Chinese Academy of Sciences, Shanghai 201210, P.R. China

[2] University of Chinese Academy of Sciences, Beijing 100049, P.R. China

[3] Shanghai Institute of Applied Physics, Chinese Academy of Sciences, Shanghai 201800, P.R. China

[4] School of Physical Science and Technology, ShanghaiTech University, Shanghai 201210, P.R. China

[5] Shanghai Synchrotron Radiation Facility, Shanghai Advanced Research Institute, Chinese Academy of Sciences, Shanghai 201210, P.R. China

*Corresponding author. Email: lisg@sari.ac.cn, lintj@sari.ac.cn, zhongls@sari.ac.cn

[#]These authors contributed equally to this work.

Table of Contents

Supplementary Methods

Supplementary Figures

Supplementary Tables

Supplementary References

Supplementary Methods

Materials

Aerosol silica (SiO_2, AEROSIL 380, purchased from Evonik Degussa China Co., Ltd.) was used as the catalyst support. Ruthenium nitrosyl nitrate (0.1414 g Ru per gram of solution, purchased from Heraeus Precious Metal Technology Co., Ltd.), $NaNO_3$, and Zr $(NO_3)_4 \cdot 5H_2O$ (purchased from Sinopharm Chemical Reagent Co., Ltd.) were used for the preparation of SiO_2-supported ruthenium catalysts.

Definitions of catalytic activity and selectivity

CO conversion was calculated according to the following equation:

$$X_{\mathrm{CO}} = \frac{CO_{inlet} - CO_{outlet}}{CO_{inlet}} \times 100\% \qquad (1)$$

Where CO_{inlet} and CO_{outlet} refer to the molar numbers of CO at the inlet and outlet, respectively.

Product selectivity was calculated according to the following equation:

$$S_i = \frac{N_i \times n_i}{\sum(N_i \times n_i)} \times 100\% \qquad (2)$$

where S_i denotes the selectivity to product i on a carbon basis, N_i is the molar fraction of product i, and n_i is the carbon number of product i. The selectivity of oxygenates was less than 1% and has been excluded from the reported product selectivity.

The ruthenium-weight-based activity (ruthenium time yield, RuTY) was calculated as following:

$$\mathrm{RuTY} = \frac{WHSV \times X_{\mathrm{CO}} \times CO\ Concentration}{22400 \times \xi_{Ru}} \qquad (3)$$

Where WHSV is the weight hourly space velocity, X_{CO} is the CO conversion, CO Concentration represents the molar concentration of CO in the feedstock, ξ_{Ru} represents the loading amount of Ru measured by XRF.

The turnover frequency (TOF) value was calculated as following:

$$\mathrm{TOF} = \frac{\mathrm{RuTY} \times \mathrm{M_{Ru}}}{\mathrm{D_{Ru}} \times 3600} \qquad (4)$$

Where M_{Ru} is the relative atomic mass of Ru (101.07 g·mol^{-1}), D_{Ru} is the dispersion of metallic Ru measured by CO chemisorption.

The space time yield of olefins product was calculated as following:

$$STY_{\mathrm{olefins}} = \frac{m_{olefins}}{(m_{cat.} \times \xi_{Ru}/101.07) \times \mathrm{t}} \tag{5}$$

Where $m_{olefins}$ is the mass of olefins product produced within a certain reaction time (t), $m_{cat.}$ is the mass of catalyst packed in the reactor.

The chain growth probability (α) was calculated according to Anderson-Schulz-Flory distribution:

$$\left(\frac{W_n}{n}\right) = (1-\alpha)^2 \alpha^{(n-1)} \tag{6}$$

where n is the carbon number of products, W_n is mass fraction of the hydrocarbons with a carbon number of n, α is chain growth probability.

The equation of (4) can be edited as:

$$\mathrm{Ln}\left(\frac{W_n}{n}\right) = (n-1)\mathrm{Ln}\alpha + 2\mathrm{Ln}(1-\alpha) \tag{7}$$

Plotting Ln(W_n/n) versus n (carbon number), and the chain growth probability (α) can be obtained by calculating the slope (Lnα).

Supplementary Catalyst Characterization

The element content of various samples was measured by an energy dispersive X-ray fluorescence (XRF, Rigaku ZSX Primus II) and ICP-OES (Varian ICP-OES 720).

The specific surface area, pore volume, and the average pore diameter of the samples were measured by N_2 physisorption on Micromeritics 2020 instrument working at –196 °C. Prior to N_2 adsorption, the samples were degassed under vacuum at 300 °C for 10 h. Brunauer-Emmett-Tellwer (BET) method was used to calculate the specific surface areas. Pore volume and pore size were determined by the Barrett-Joyner-Halenda (BJH) method.

Transmission electron microscopy (TEM) and high-solution transmission electron microscopy (HRTEM) measurements were conducted on a FEI Tecnai G2 F20 S-TWIN equipment with 200 kV accelerating voltage. The nanoparticle

size distribution for each sample was determined using at least 200 nanoparticles. The size distribution of metallic Ru was analyzed based on the statistics of over 200 particles. The dispersion of metallic Ru nanoparticles (D_{TEM}) was further calculated from the following equation:

$$D_{TEM} = \frac{6V/a}{d} = \frac{6Mn_s}{\rho N_A d} \quad (7)$$

where V denotes the volumes of a Ru atom in the bulk of the metal (Å^3), a denotes the specific surface area of a Ru atom (Å^2), d denotes the mean particle size (nm), M denotes the Ru atomic mass (g·mol^{-1}), n_s denotes the mean number of atoms in the exposed surface area (m^{-2}), ρ denotes the mass density of metallic Ru (g·cm^{-3}), and N_A is Avogadro constant (6.02 × 10^{23} mol^{-1}). For *hcp* Ru exposed (001) surface, the above equation can be converted to

$$D_{TEM} = \frac{1.12}{d} \quad (8)$$

Temperature programmed reduction with hydrogen (H_2-TPR), temperature programmed desorption of H_2 (H_2-TPD) and temperature-programmed surface reaction (TPSR) of CO measurements were all carried out on a chemisorption apparatus (Micromeritics 2920 Auto Chem II) with a thermal conductivity detector (TCD) and MKS Cirrus 2 mass spectrometer (MS). Before TPR measurement, the samples were pretreated in He flow at 120 °C for 60 min, then cooled down to 50 °C. The reactor was heated to 800 °C at a heating rate of 10 °C·min^{-1} in 5 vol% H_2 (balance Ar, 30 mL·min^{-1}). The signal of H_2 consumption was monitored by TCD. The H_2 temperature programmed desorption (H_2-TPD) measurements were performed on fresh catalysts. The 100 mg of sample was first reduced at 450 °C for 2 h under flowing H_2. H_2 adsorption was employed at 50 °C for 30 min by passing H_2 with a flow rate of 30 mL·min^{-1}. The TPD signal was recorded by TCD and the signals of m/z = 2 (H_2) was monitored by mass spectrometer with linear temperature increase up to 800 °C at a ramp rate of 10 °C·min^{-1}. For the temperature-programmed surface reaction of CO (CO-TPSR), the sample was pretreated in a H_2 flow at 450 °C for 2 h and then flushing with He for 30 min. The temperature was cooled down to 50 °C

and CO flow was switched to realize the saturated CO adsorption. The sample was heated up to 500 °C at 10 °C·min^{-1} in flowing of H_2 flow, and the signals of m/z=16 (CH_4) was monitored by mass spectrometer.

Hydrogen spillover detection by the WO_3 powder experiment was performed in a quartz reaction tube at 100 °C. 1 g of WO_3 and 0.02 g of catalyst samples were placed in a quartz reaction tube and held in place using silica wool. Then, the tube temperature was controlled to be 100 °C in a furnace, and H_2 was introduced into the reaction tube at a consistent rate of 50 mL·min^{-1}. After 60 min, the H_2 flow was switched to Ar and cooled to room temperature. Finally, the reaction tube was removed, and color changes in the WO_3 powder were observed and recorded.

Supplementary Figures

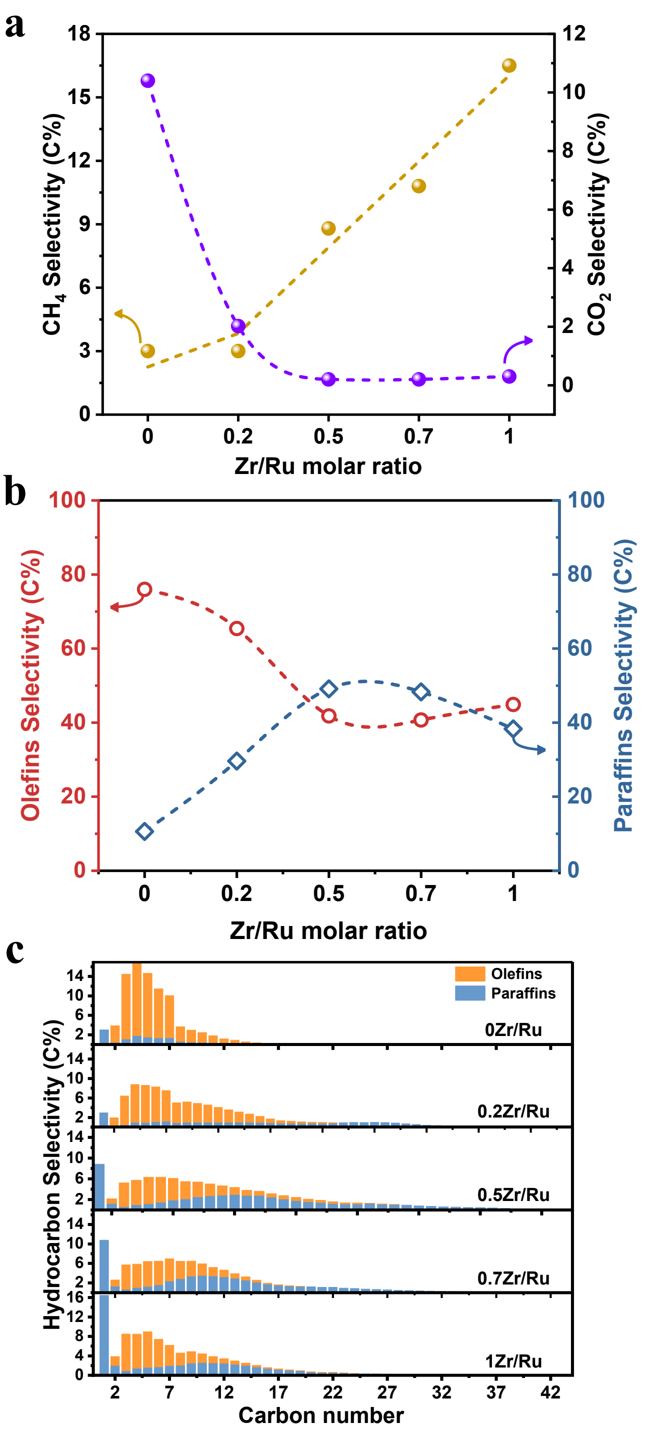

Supplementary Fig. 1. Catalytic performance of *x*Zr/Ru catalysts under the same reaction conditions (T = 260 °C, H_2/CO = 2, P = 5 bar and WHSV = 3000 mL·g^{-1}·h^{-1}). (**a, b**) Product selectivity and (**c**) hydrocarbon product distributions of various *x*Zr/Ru catalysts.

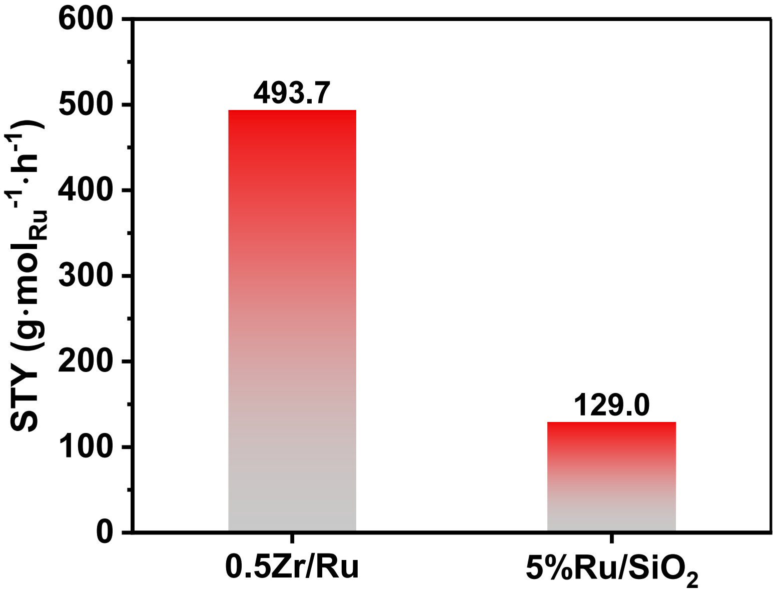

Supplementary Fig. 2. The comparison of olefins STY over 0.5Zr/Ru and 5%Ru/SiO_2 catalysts at similar CO conversion level (T = 260 °C, P = 5 bar, H_2/CO=2, and WHSV = 3000 – 12000 mL·g^{-1}·h^{-1}).

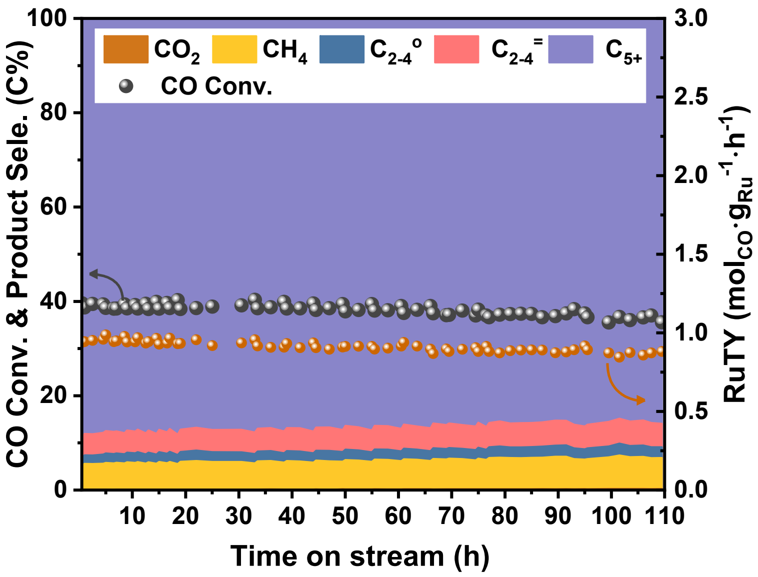

Supplementary Fig. 3 Stability test for 0.5Zr/Ru catalyst (T = 260 °C, H_2/CO = 2, P = 5 bar and WHSV = 3000 mL·g^{-1}·h^{-1}).

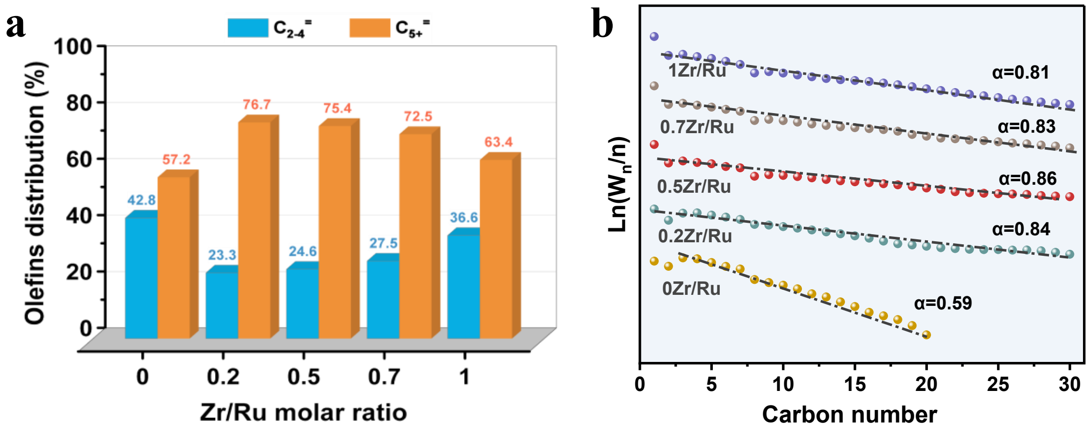

Supplementary Fig. 4. Olefins distribution (**a**) and Anderson-Schulz-Flory plots (**b**) over various *x*Zr/Ru catalysts under similar CO conversion level (T = 260 °C, H_2/CO = 2, P = 5 bar and WHSV = 3000 – 12000 mL·g^{-1}·h^{-1}).

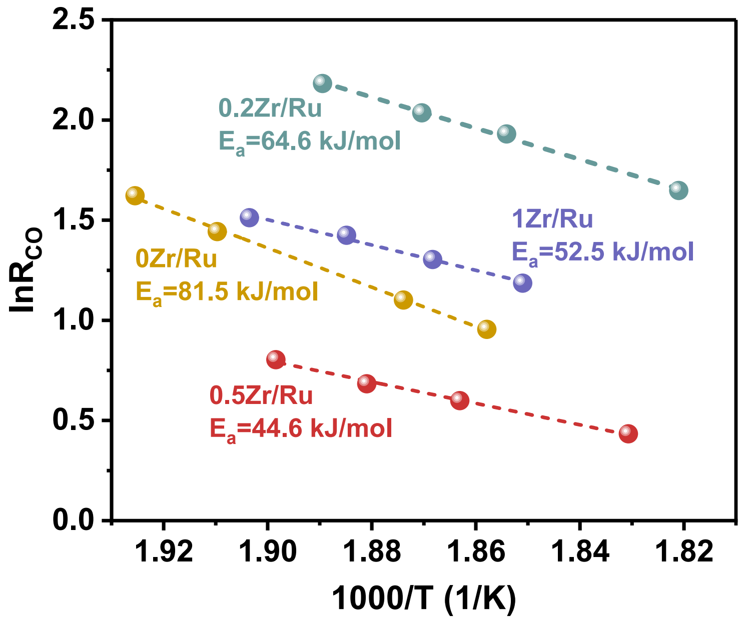

Supplementary Fig. 5. Arrhenius plots of the *x*Zr/Ru catalysts for CO conversion.

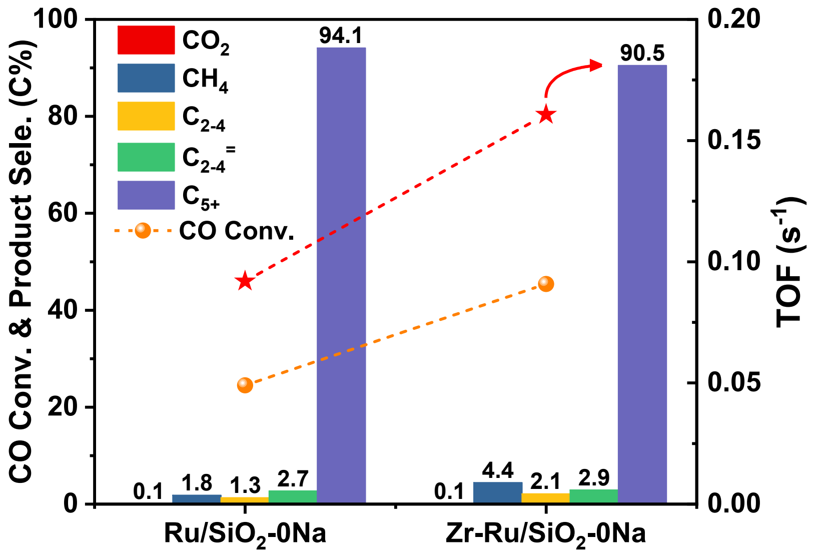

Supplementary Fig. 6. Effect of the Zr promoter on the catalytic performance of Na-free catalysts under the same reaction conditions (220 °C, 10 bar, 3000 mL·g^{-1}·h^{-1}, and H_2/CO=2).

Notes:

It was found that the addition of Zr promoter notably enhanced the catalytic activity, as evidenced by the increase of CO conversion from 24.5% for Ru/SiO_2-0Na to 45.4% for Zr-Ru/SiO_2-0Na under the same reaction conditions (220 °C, 10 bar, 3000 mL·g^{-1}·h^{-1}, and H_2/CO=2). Moreover, it moderately improves CH_4 selectivity from 1.8% to 4.4% and decreases C_{5+} selectivity from 94.1% to 90.5%. Due to the low water-gas shift activity of Ru-based catalysts, CO_2 selectivity remains almost negligible for both Ru/SiO_2-0Na and Zr-Ru/SiO_2-0Na catalysts. The intrinsic activity was further calculated as displayed in Supplementary Fig. 6 with 0.092 s^{-1} and 0.161 s^{-1} for Ru/SiO_2-0Na and Zr-Ru/SiO_2-0Na, respectively. It is evident that introducing the Zr promoter significantly enhances the intrinsic activity of the Ru/SiO_2 catalyst.

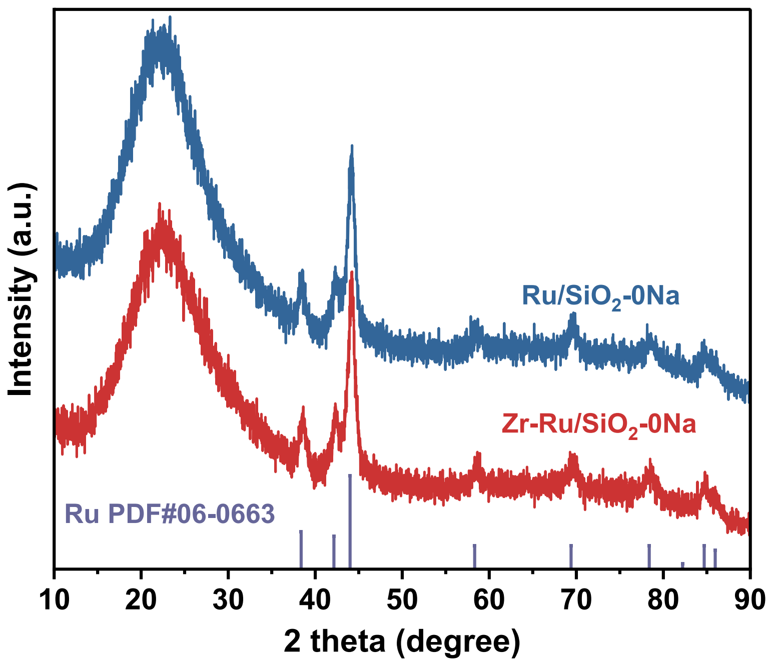

Supplementary Fig. 7. XRD patterns for the reduced catalysts of Ru/SiO_2-0Na and Zr-Ru/SiO_2-0Na.

Notes:

XRD patterns of the reduced catalysts without Na promoter were shown in Supplementary Fig. 7. It can be observed that adding the Zr promoter does not affect the phase of the catalysts, which remains metallic Ru (JCPDS, 06-0663). Furthermore, no diffraction peaks for the crystalline phase of the ZrO_2 promoter were observed.

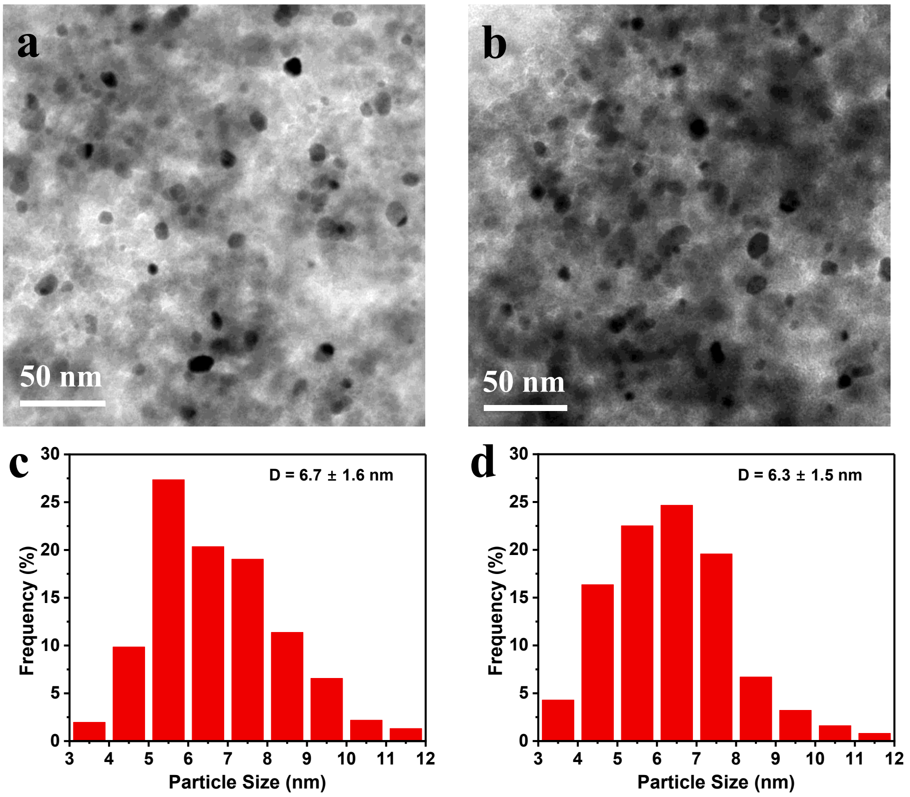

Supplementary Fig. 8. TEM images and the corresponding particle size distribution for the Na-free reduced catalysts. (a, c) Ru/SiO_2-0Na, (b, d) Zr-Ru/SiO_2-0Na.

Notes:

Comparison of the average particle sizes of metallic Ru nanoparticles measured by TEM for all reduced samples indicates that the ZrO_2 loading exhibits little influence on the size of metallic Ru nanoparticles (Supplementary Fig. 8). The dispersion of metallic Ru nanoparticles, calculated based on the average particle size obtained from the TEM results, is determined to be 16.7% and 17.7% for Ru/SiO_2-0Na and Zr-Ru/SiO_2-0Na, respectively.

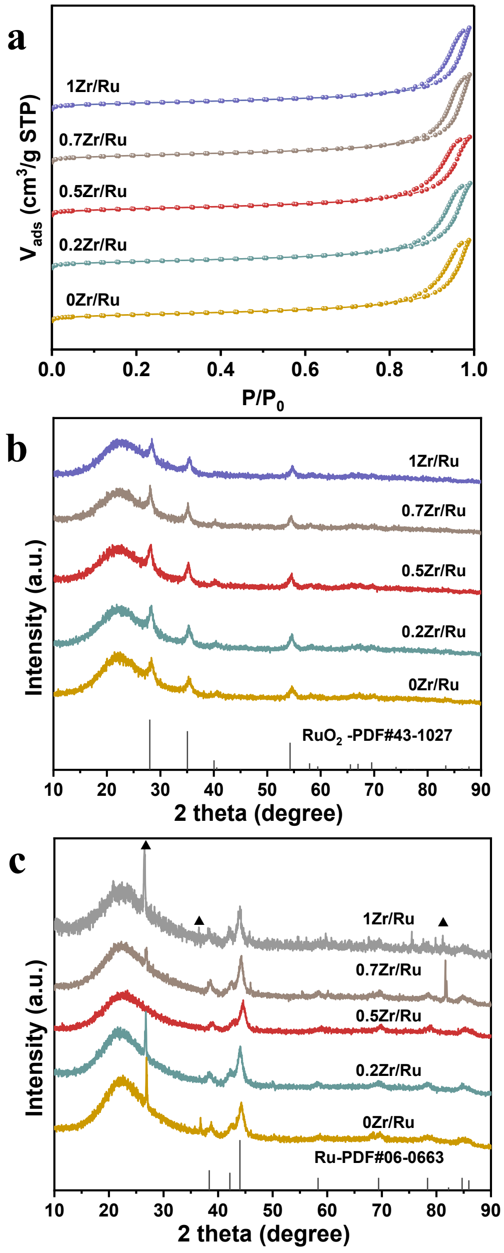

Supplementary Fig. 9. (**a**) N_2 adsorption-desorption isotherms for various *x*Zr/Ru samples; (**b**) XRD patterns for the fresh catalysts; (**c**) XRD patterns for the spent catalysts. (▲ represents the diffraction peaks of diluent SiO_2.)

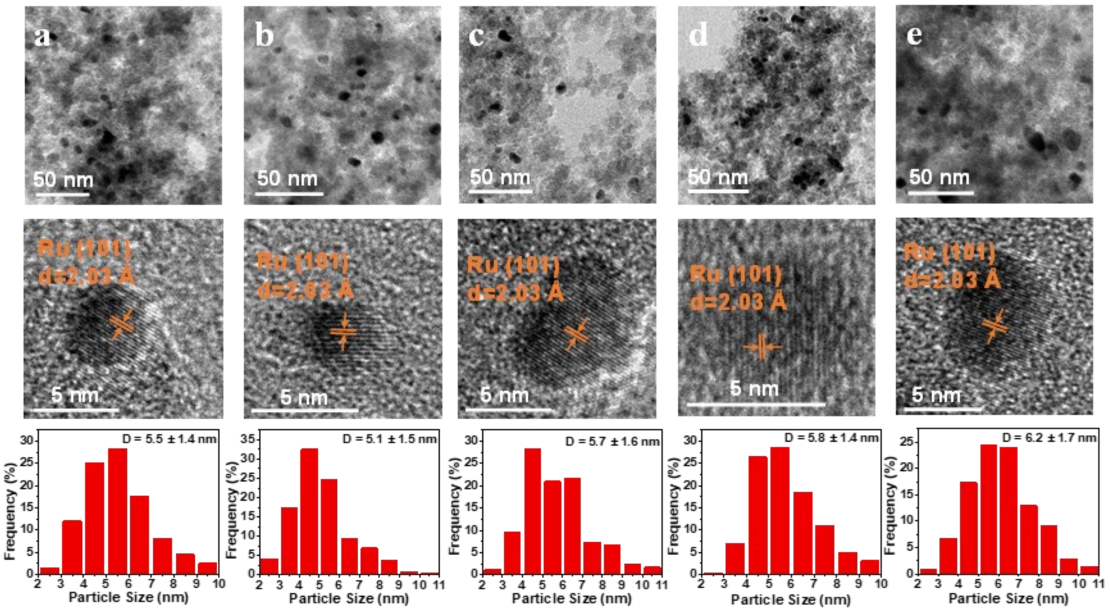

Supplementary Fig. 10. (HR)TEM images and the corresponding particle size distribution for the reduced *x*Zr/Ru catalysts. (**a**) 0Zr/Ru, (**b**) 0.2Zr/Ru, (**c**) 0.5Zr/Ru, (**d**) 0.7Zr/Ru, (**e**) 1Zr/Ru.

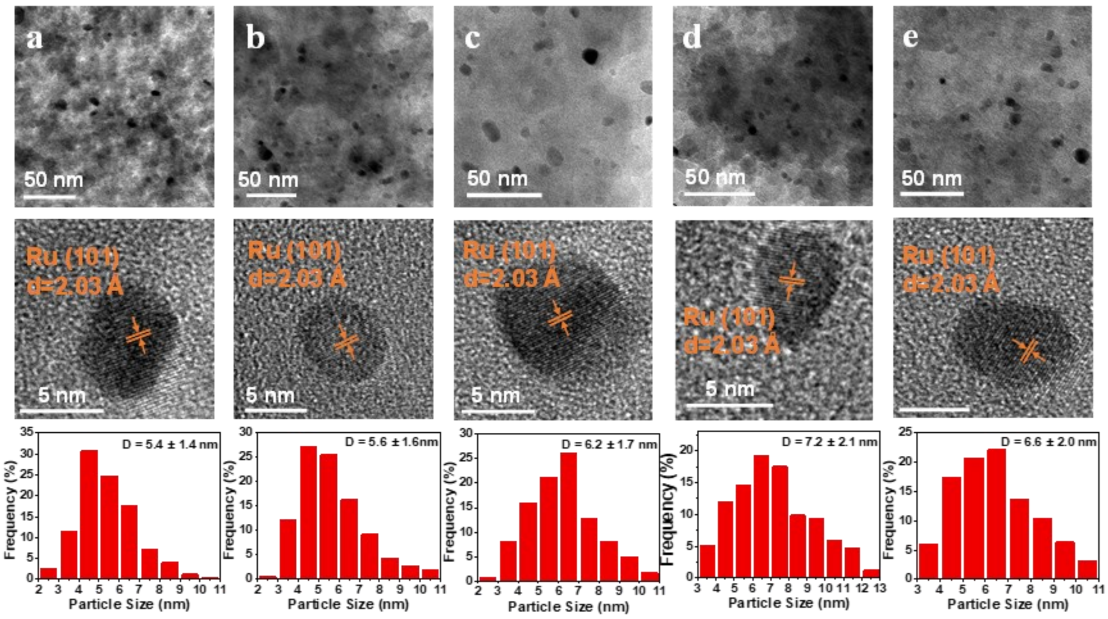

Supplementary Fig. 11. (HR)TEM images and the corresponding particle size distribution for the spent *x*Zr/Ru catalysts. (**a**) 0Zr/Ru, (**b**) 0.2Zr/Ru, (**c**) 0.5Zr/Ru, (**d**) 0.7Zr/Ru, (**e**) 1Zr/Ru.

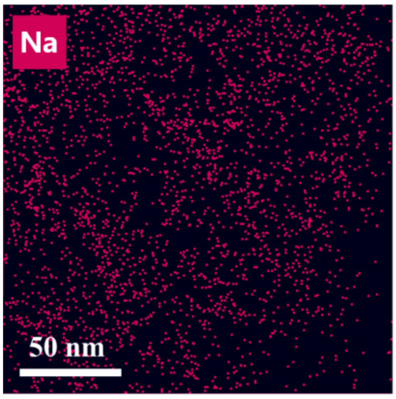

Supplementary Fig. 12. Na elemental mapping of the reduced 0.5Zr/Ru sample determined by STEM-EDS.

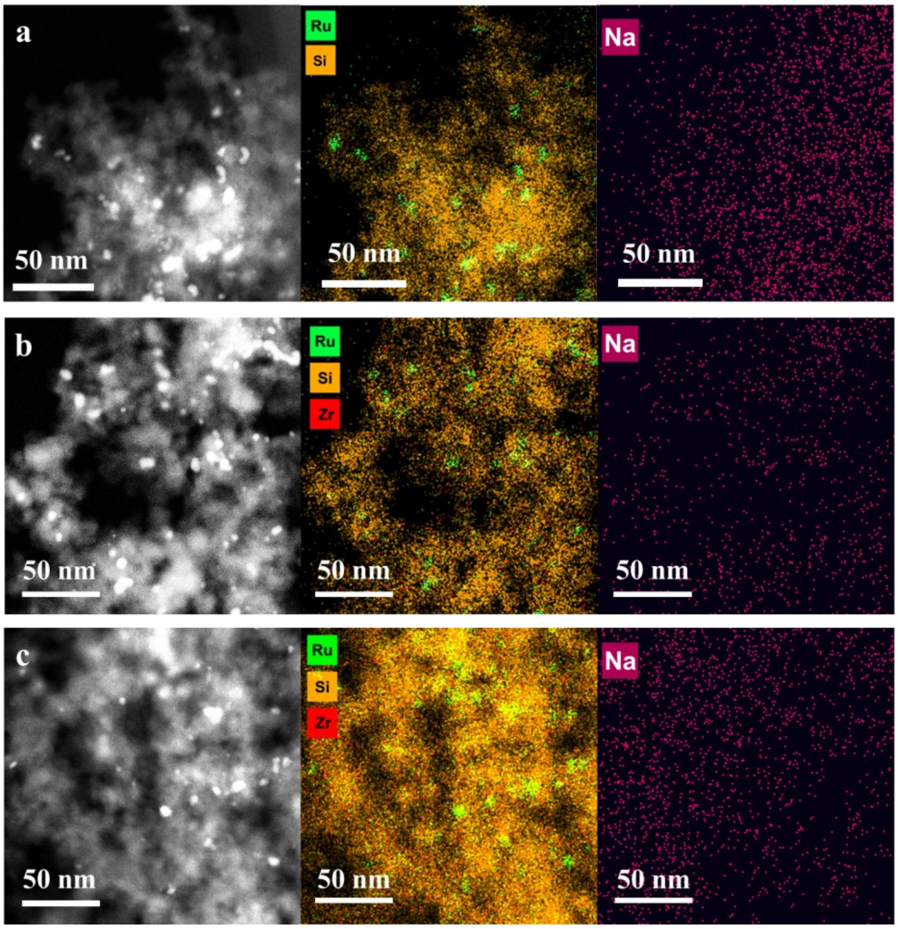

Supplementary Fig. 13. HAADF-STEM images and STEM-EDS elemental mapping of the reduced (**a**) 0Zr/Ru, (**b**) 0.2Zr/Ru and (**c**) 1Zr/Ru catalysts. Ru is depicted in green, Si in yellow, Zr in red, and Na in purple.

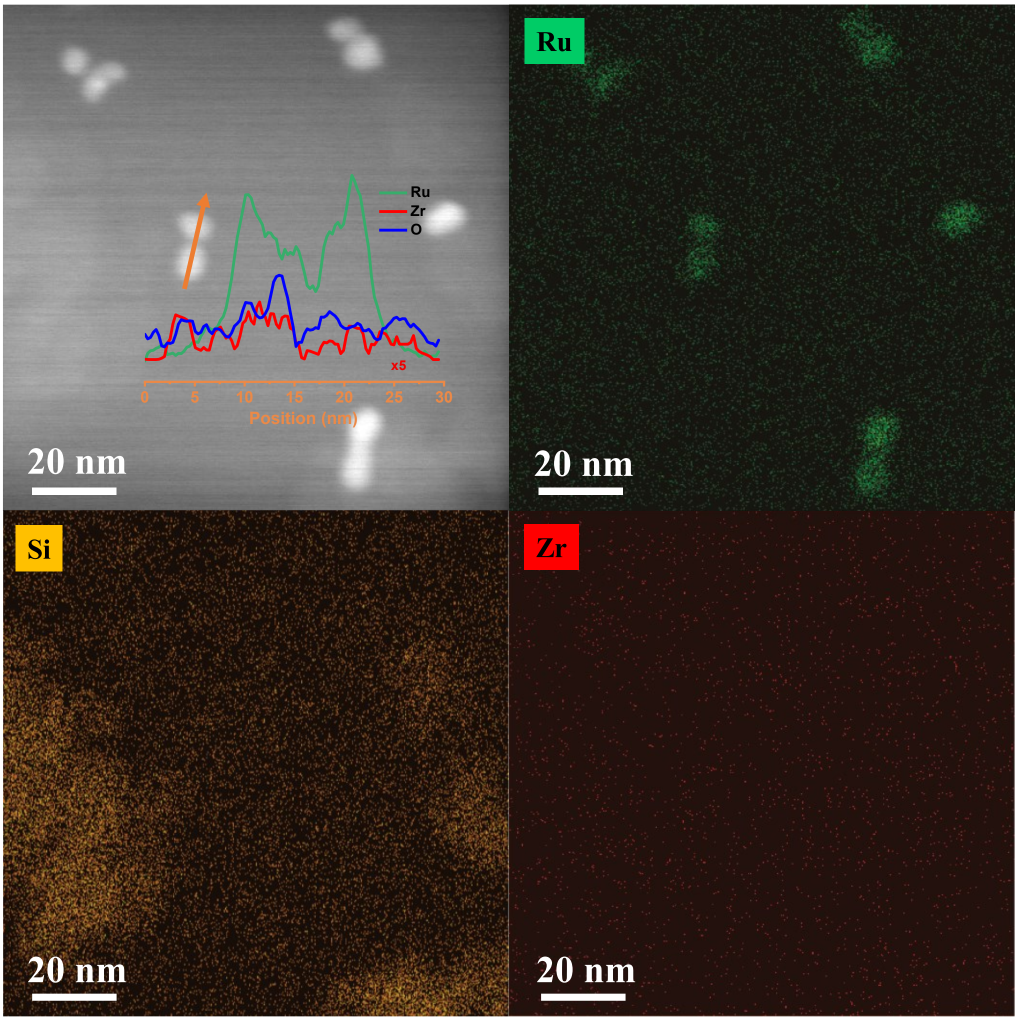

Supplementary Fig. 14. HAADF-STEM images and STEM-EDS elemental mapping of the spent 0.5Zr/Ru catalyst. Ru is depicted in green, Si in yellow, Zr in red. The orange arrow represents the line-scanning direction. The related STEM-EDS line-scanning results are displayed in the insets.

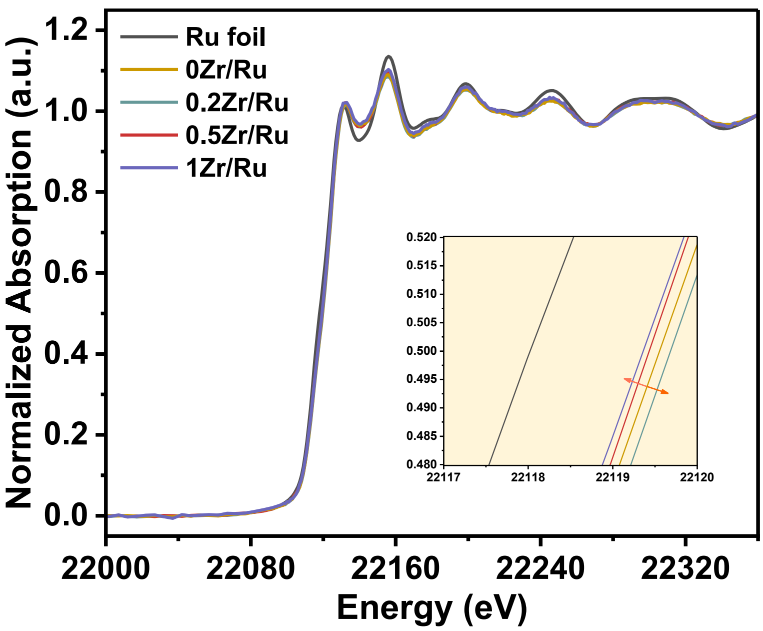

Supplementary Fig. 15. Ru K-edge XANES spectra of the reduced *x*Zr/Ru samples.

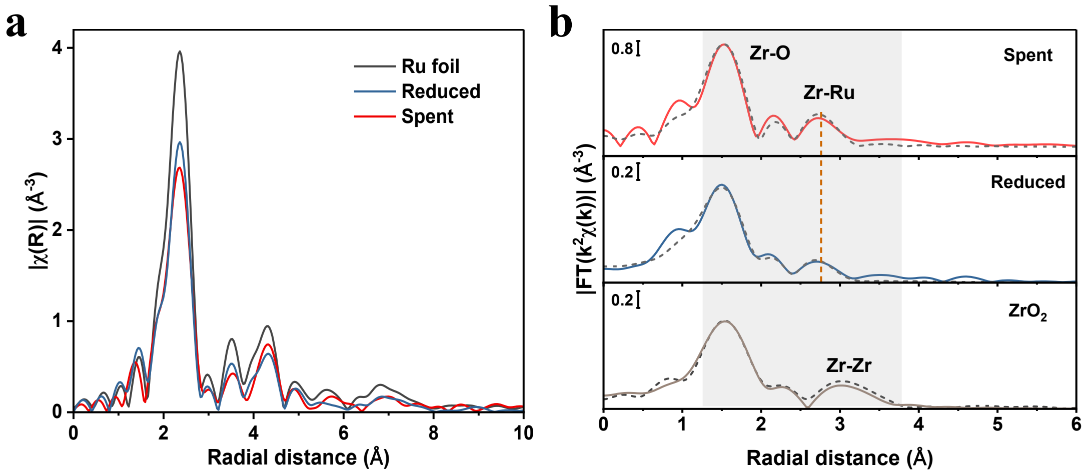

Supplementary Fig. 16. (**a**) Normalized Ru K-edge k_2-weighted EXAFS spectra of the reduced and spent 0.5Zr/Ru samples. (**b**) Experimental (colored lines) and best fit (dashed black lines) FT-EXAFS spectra for the reduced and spent 0.5Zr/Ru samples measured at the Zr K-edge.

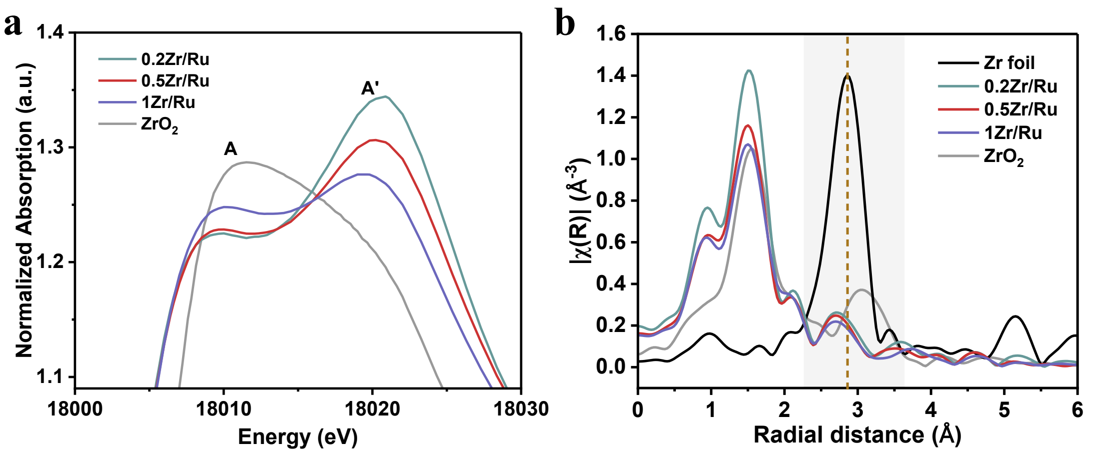

Supplementary Fig. 17. Structure determination of the Zr promoter. (**a**) XANES partial enlarged detail enhancing the white-line peak feature for the reduced *x*Zr/Ru (*x* = 0.2, 0.5, and 1) samples. (**b**) Normalized Zr K-edge k_2-weighted EXAFS spectra of the *x*Zr/Ru samples.

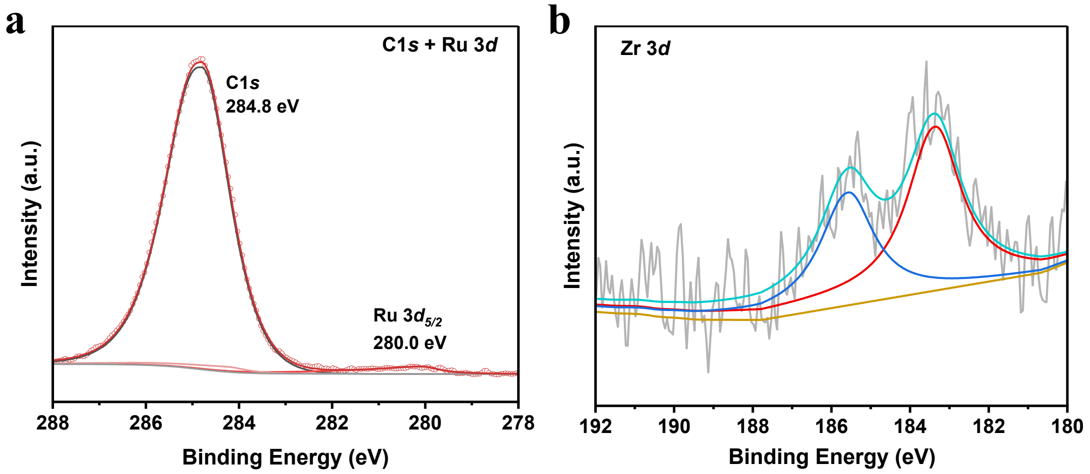

Supplementary Fig. 18. XPS spectra of the Ru 3*d* (a) and Zr 3*d* (b) line for the spent 0.5Zr/Ru catalyst.

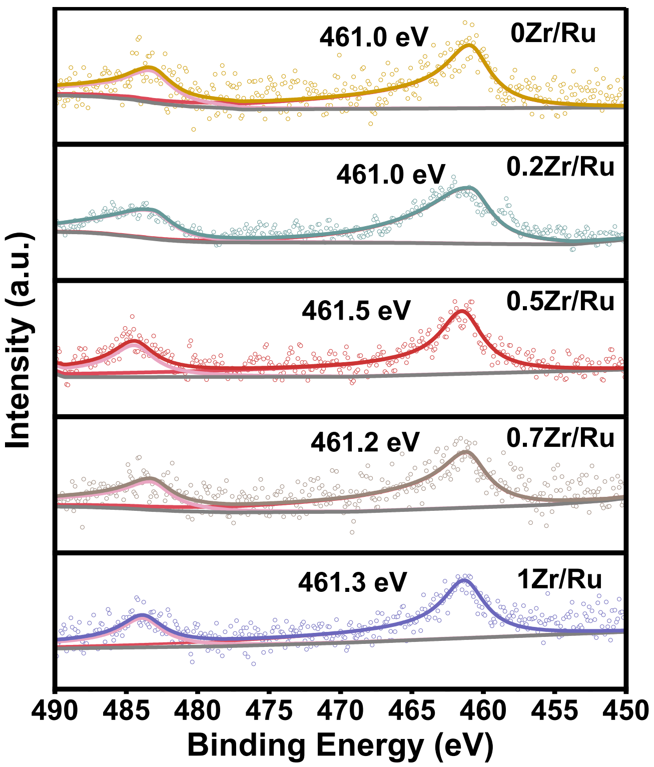

Supplementary Fig. 19. XPS spectra of various reduced catalysts at the Ru 3*p* level.

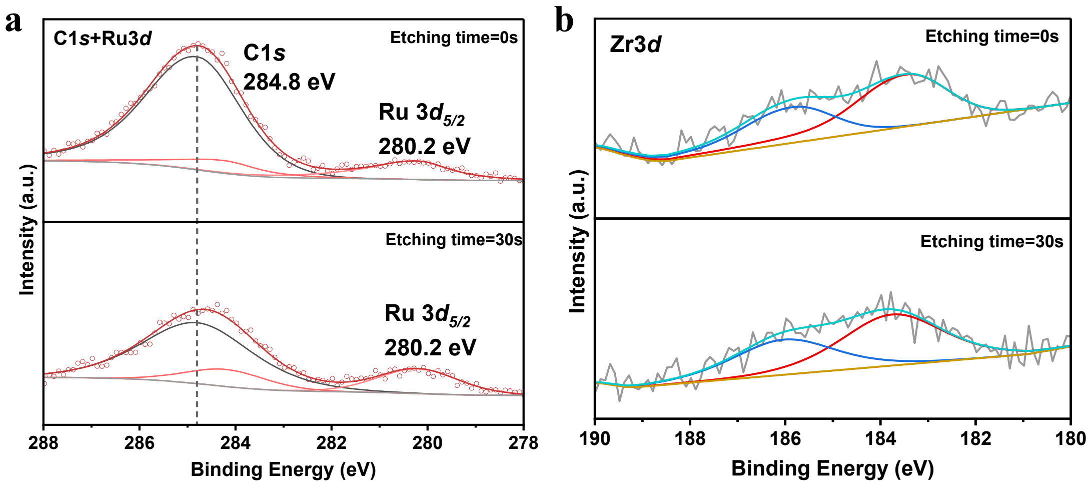

Supplementary Fig. 20. XPS spectra of the C1*s*, Ru 3*d* (**a**) and Zr 3*d* (**b**) levels for the reduced 0.5Zr/Ru catalyst after XPS sputter etching.

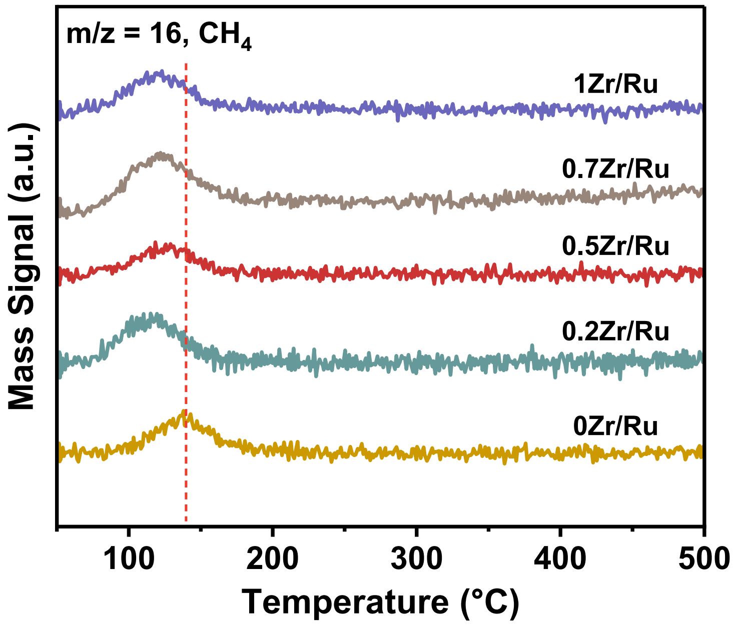

Supplementary Fig. 21. CO-TPSR profiles for various *x*Zr/Ru (*x* = 0, 0.2, 0.5, 0.7, 1) samples.

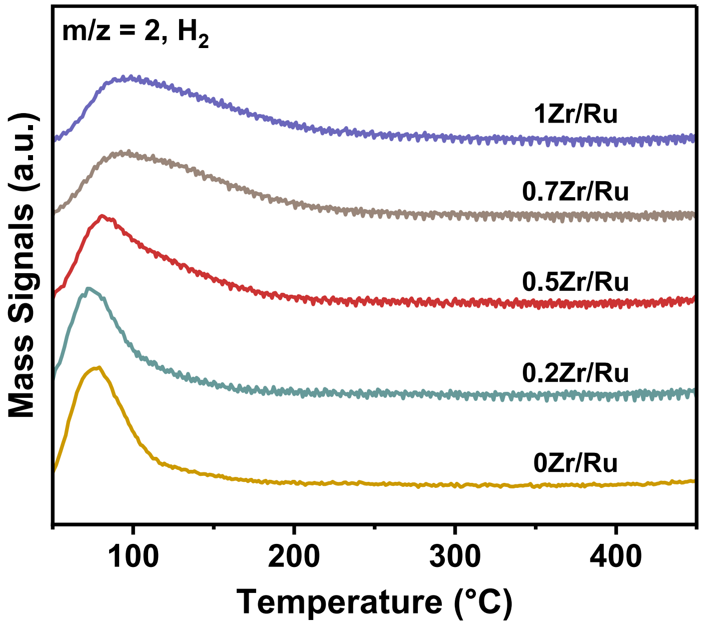

Supplementary Fig. 22. H_2-TPD profiles for various *x*Zr/Ru (*x* = 0, 0.2, 0.5, 0.7, 1) catalysts.

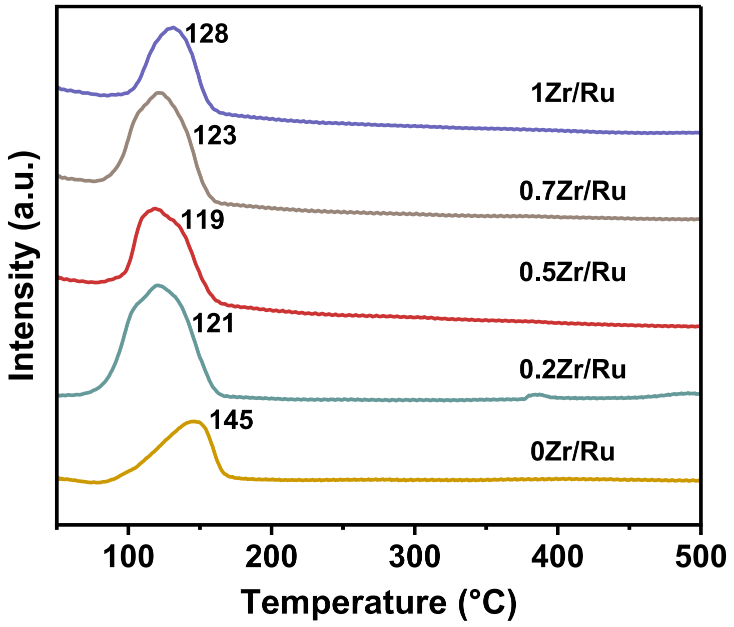

Supplementary Fig. 23. H_2-TPR profiles for various *x*Zr/Ru (*x* = 0, 0.2, 0.5, 0.7, 1) catalysts.

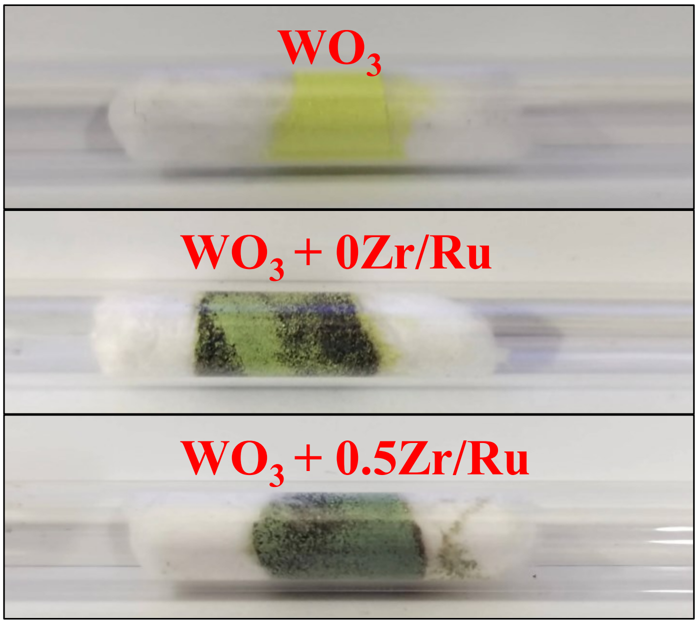

Supplementary Fig. 24. Photographs of the samples made with 1 g WO_3 mixed with 0.02 g 0Zr/Ru or 0.5Zr/Ru catalysts after treatment with H_2 at 100 °C for 1 h.

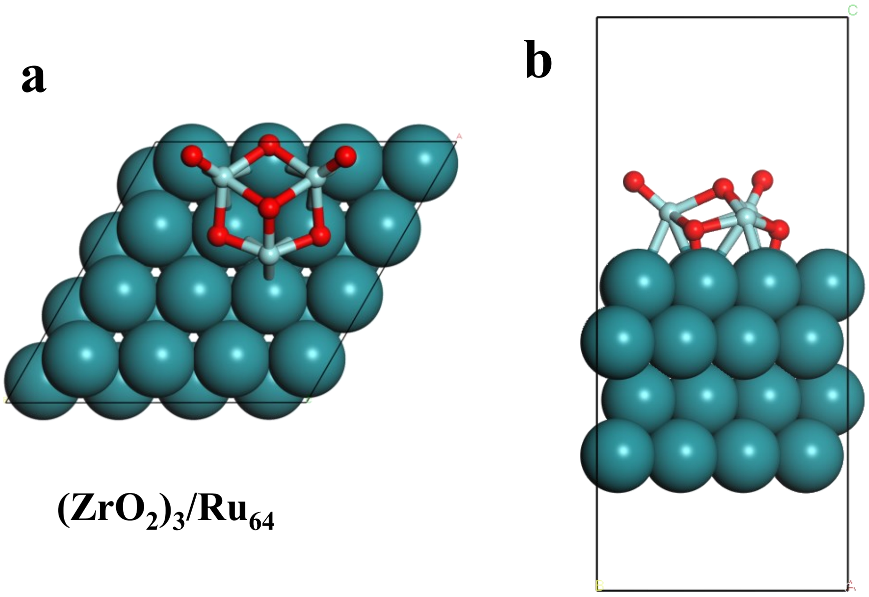

Supplementary Fig. 25. The top (**a**) and side (**b**) view of optimized structure for Zr-O-Ru interfacial structure. Ru (dark green spheres), Zr (light green spheres), and O (red spheres) atoms are shown.

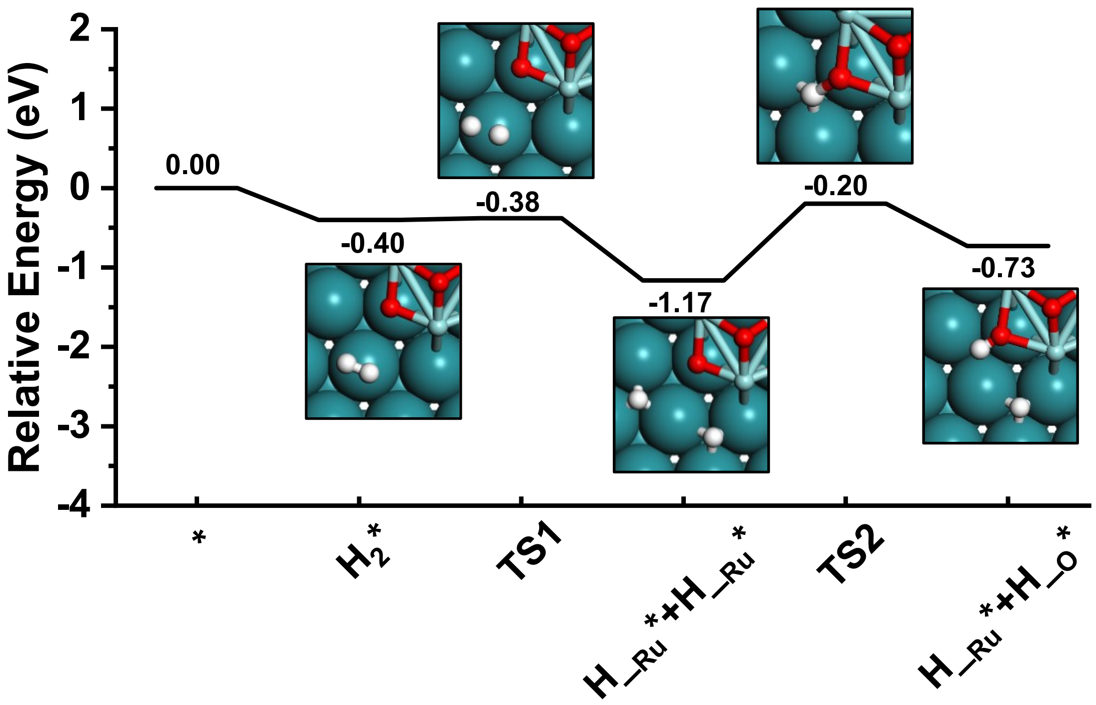

Supplementary Fig. 26. Energy profiles and the corresponding structures in the process of H_2 dissociation and hydrogen atoms spillover on $(ZrO_2)_3$/Ru (001). Ru (dark green spheres), Zr (light green spheres), O (red spheres), and H (white spheres) atoms are shown.

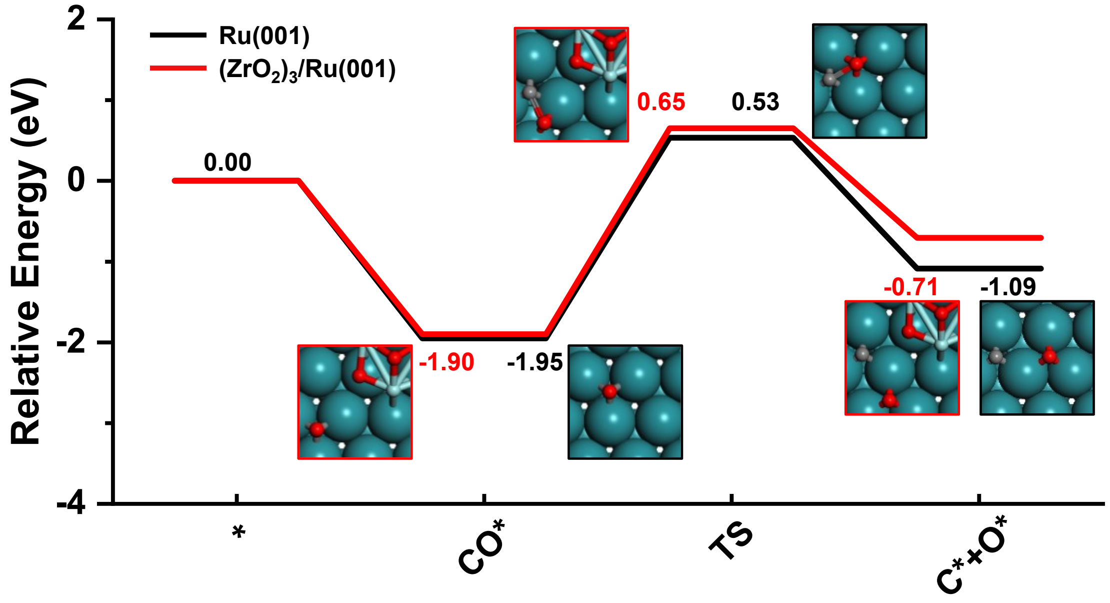

Supplementary Fig. 27. Energy profiles and the corresponding structures in the process of CO direct dissociation on Ru (001) and $(ZrO_2)_3$/Ru (001) surface, respectively. Ru (dark green spheres), Zr (light green spheres), O (red spheres), and C (gray spheres) atoms are shown.

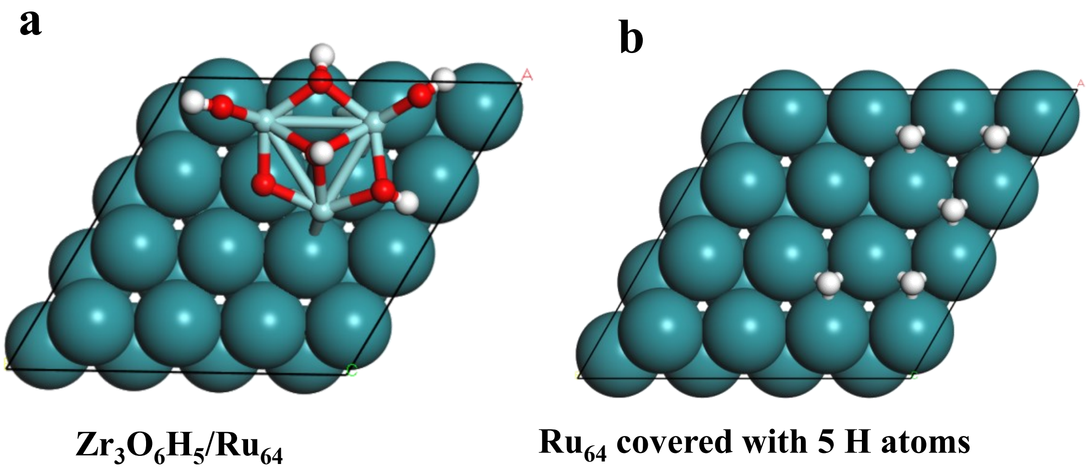

Supplementary Fig. 28. The top view of optimized structure for Zr-O-Ru interfacial structure (**a**) and pure metallic Ru surface (**b**) with higher H coverage. Ru (dark green spheres), Zr (light green spheres), O (red spheres) atoms, and H (white spheres) atoms are shown.

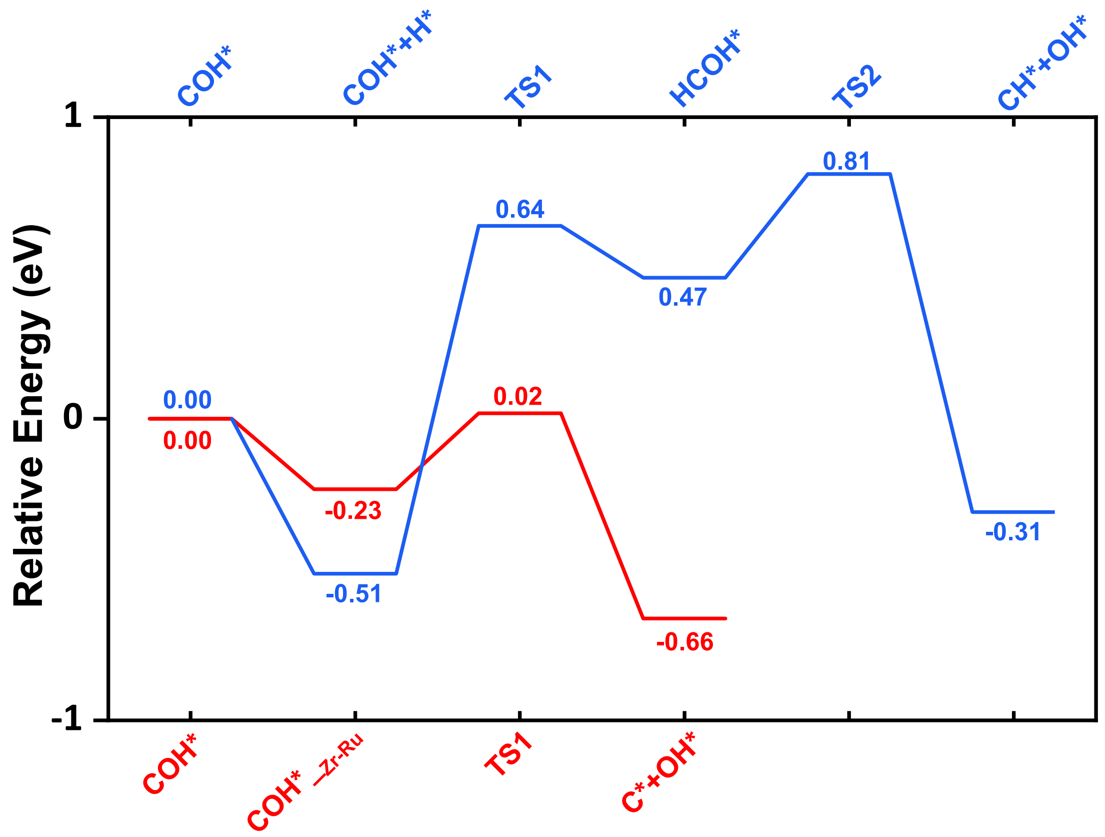

Supplementary Fig. 29. Energy profiles of different intermediates and COH* dissociation over $Zr_3O_6H_5$/Ru (001) surface. Red solid line represents the energy profile of COH* dissociation to C* and OH* pathway and blue solid line represents the energy profile of COH* hydrogenation to HCOH* dissociation pathway.

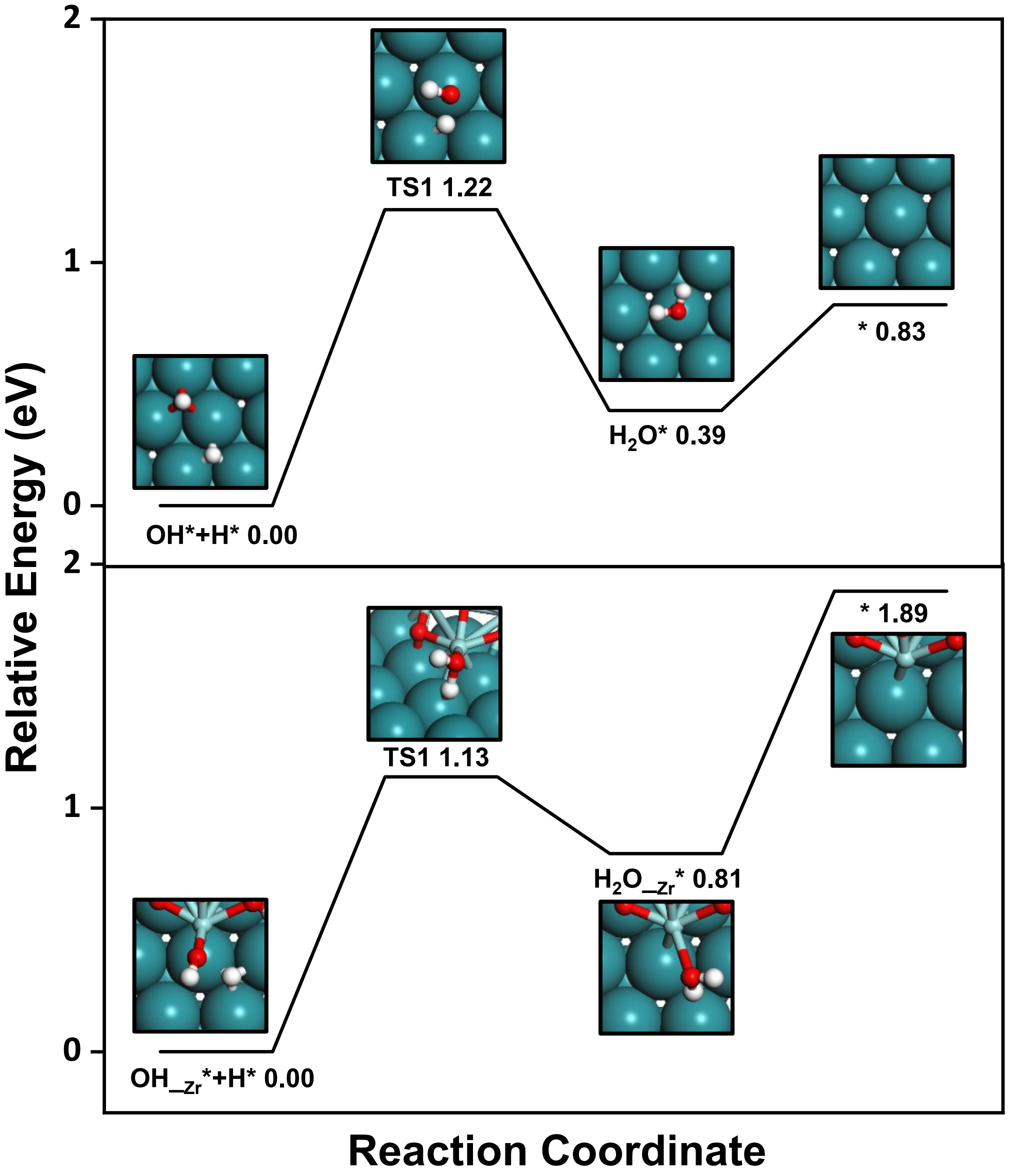

Supplementary Fig. 30. Energy profiles for the hydrogenation of the OH* species to H_2O molecules and the further desorption of H_2O molecules at the Ru sites of Ru (001) (top)and the Zr sites of $Zr_3O_6H_5$/Ru (001) (bottom).

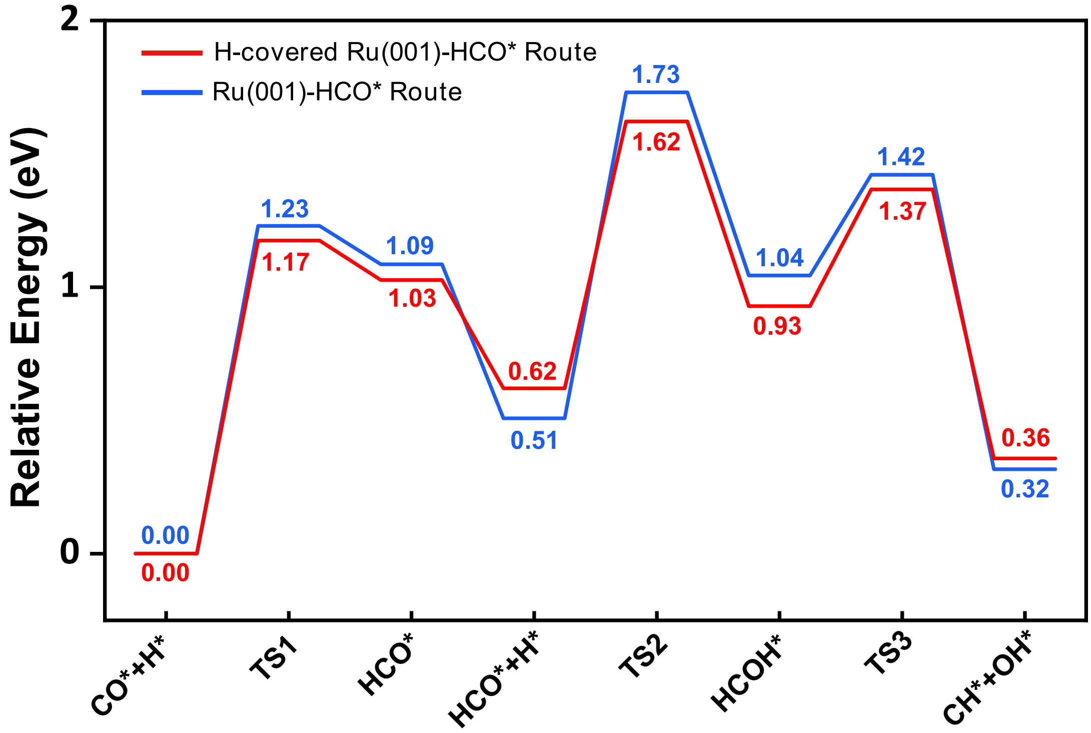

Supplementary Fig. 31. Energy profiles of H-assisted CO dissociation via the HCO* route over the Ru (001) and H-covered Ru (001) surfaces.

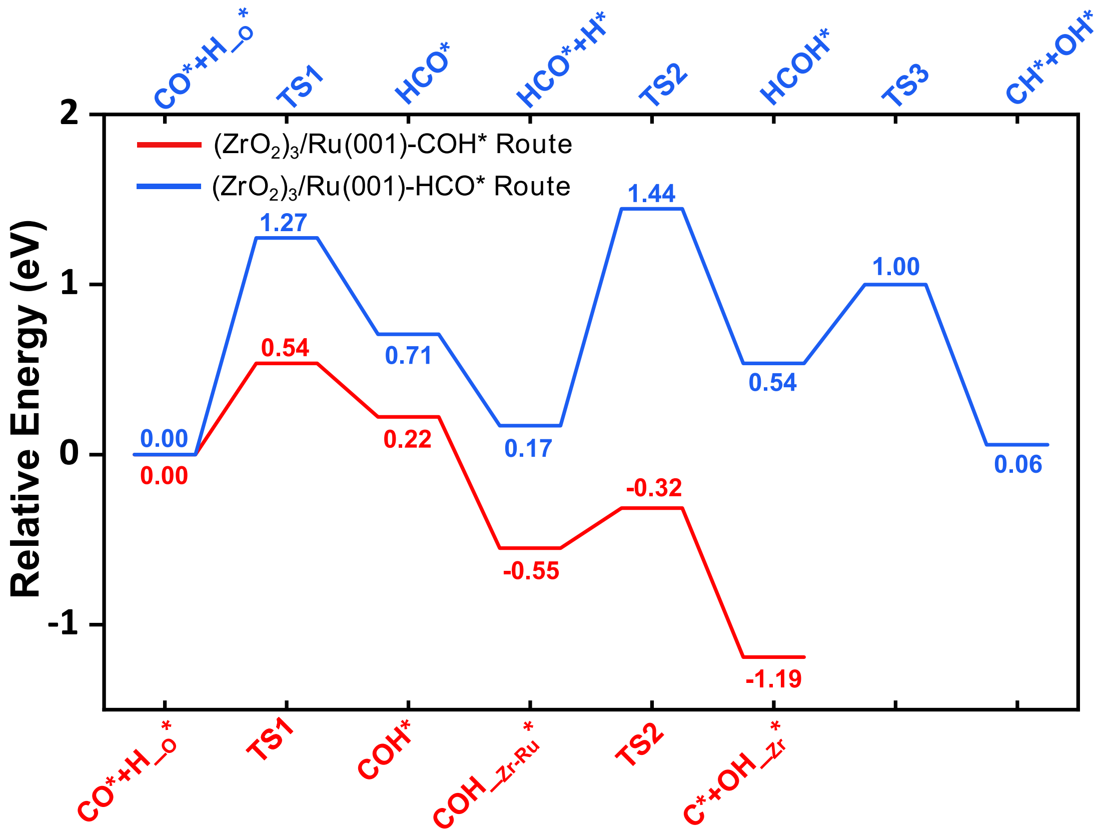

Supplementary Fig. 32. Energy profiles of H-assisted CO dissociation via different intermediates (HCO* and COH*) over the $(ZrO_2)_3$/Ru (001) surface.

Supplementary Tables

Supplementary Table 1. Comparison of the catalytic activity of various supported Ru-based catalysts for FTS reaction.

Entry	Catalyst	T (°C)	P (MPa)	RuTY ($mol_{CO}·g_{Ru}^{-1}·h^{-1}$)	TOF (s^{-1})	Ref.
1	0.5Zr/Ru	260	0.5	0.955	0.316	This work
2	Na-Ru/SiO_2	260	1	0.472	0.126	[1]
3	Ru/TiO_2-450R	160	2	0.473	0.039	[2]
4	Ru/TiO_2(R)	250	0.1	0.547	-	[3]
5	Ru/Al_2O_3-PHR	150	3	0.129	0.006	[4]
6	Ru/Al_2O_3-10Cl	250	4.04	0.333	0.03	[5]
7	Ru/C	230	2	0.077	-	[6]
8	Ru@Si/Al-50	270	2	0.404	-	[7]
9	Ru/meso-ZSM-5	260	2	0.507	0.067	[8]
10	Ru/meso-beta	260	2	0.531	0.071	[9]
11	Ru/HB-S	260	1	-	0.129	[10]
12	Ru/CNT	260	2	0.583	0.193	[11]
13	Ru@MHCS	250	1	0.342	0.023	[12]
14	Ru/TiO_2-500-H	220	2	-	0.047	[13]
15	Ru-in/TNT	240	2	0.216	-	[14]
16	10Ru/G100	240	1.5	0.689	-	[15]
17	3%Ru/CNT	230	1	0.32	-	[16]

Supplementary Table 2. Elemental composition of various fresh catalysts determined by XRF and ICP-OES.

Catalyst	Ru content (wt.%)[a]	Zr content (wt.%)[a]	Na content (wt.%)[b]	Ru content (wt.%)[b]	Zr content (wt.%)[b]	Zr/Ru molar ratio[b]
0Zr/Ru	1.86	-	0.27	1.93	-	-
0.2Zr/Ru	1.84	0.31	0.25	1.90	0.41	0.24
0.5Zr/Ru	1.87	0.82	0.26	2.00	0.91	0.51
0.7Zr/Ru	1.90	1.24	0.27	2.10	1.59	0.84
1Zr/Ru	1.98	1.93	0.38	1.87	2.03	1.24

a: Determined by XRF. b: Determined by ICP-OES.

Supplementary Table 3. Textural properties of various *x*Zr/Ru catalysts.

Catalysts	BET surface area ($m^2 \cdot g^{-1}$)	Pore volume ($cm^3 \cdot g^{-1}$)	Average pore size (nm)
0Zr/Ru	285.4	1.2	17.0
0.2Zr/Ru	280.0	1.3	17.7
0.5Zr/Ru	286.0	1.2	16.4
0.7Zr/Ru	287.7	1.3	17.6
1Zr/Ru	280.6	1.2	17.4

Supplementary Table 4. Physicochemical properties of various *x*Zr/Ru catalysts.

Catalysts	Crystallite size (nm)[a]			Particle size (nm)[b]		D_{TEM} (%)[b]	D_{CO} (%)[c]
	Fresh	Reduced	Spent	Reduced	Spent		
0Zr/Ru	8.5	6.0	7.7	5.5	5.4	18.7	11.3
0.2Zr/Ru	9.1	7.0	8.6	5.1	5.6	16.0	9.3
0.5Zr/Ru	9.7	8.4	8.0	5.8	6.3	13.3	8.5
0.7Zr/Ru	10.1	8.3	8.9	5.8	7.2	13.5	7.9
1Zr/Ru	8.9	8.0	8.5	6.2	6.6	14.0	5.2

[a] Calculated by XRD. [b] Determined by TEM. [c] Determined by CO chemisorption.

Supplementary Table 5. Results from Zr K-edge EXAFS fit of the *x*Zr/Ru samples using Zr foil and monoclinic ZrO_2 structure as reference.

Sample	Shell	CN[a]	[b]R(Å)	[c]σ^2(Å^2)	[d]ΔE_0(eV)	[e]R factor
Zr foil	Zr-Zr	12.0	2.0	-	-	-
0.2Zr/Ru	Zr-O	7.8±0.3	2.07	0.009	-4.6±1.4	0.008
	Zr-Ru	2.8±0.7	2.99	0.014	7.9±2.9	
0.5Zr/Ru	Zr-O	7.2±0.3	2.06	0.01	-6.5±1.5	0.013
	Zr-Ru	2.5±0.6	2.98	0.014	5.2±2.7	
1Zr/Ru	Zr-O	6.8±0.4	2.08	0.01	-5.5±1.9	0.011
	Zr-Ru	2.4±0.8	2.99	0.015	4.9±3.6	
0.5Zr/Ru-spent	Zr-O	6.3±0.3	2.06	0.009	5.4±1.8	0.010
	Zr-Ru	2.1±1.1	2.98	0.011	4.6±2.9	
ZrO_2	Zr-O	7.0	2.22	0.001	-2.10	0.017
	Zr-Zr	12.0	3.70	0.018		

[a] CN is the coordination number for the absorber-backscatter pair.

[b] R is the average interatomic distance.

[c] σ^2 is the Debye-Waller factor.

[d] ΔE_0 is the inner potential correction.

Supplementary Table 6. Relative energies (eV) of intermediates involved in H-assisted CO dissociation over Ru (001) and $Zr_3O_6H_5$/Ru (001) surfaces.

	Species	Ru (001)	Species	$Zr_3O_6H_5$/Ru (001)
	CO+H	0.00	CO+H_O	0.00
COH* Route	COH	0.66	COH	-0.12
	C+OH	1.26	COH_Zr-Ru	-0.35
			C+OH_Zr	-0.78
HCO* Route	HCO	1.09	HCO	0.09
	HCO+H	0.51	HCO+H	-0.39
	HCOH	1.04	HCOH	0.32
	CH+OH	0.32	CH+OH	-0.42

Supplementary Table 7. Adsorption energy (eV) of the OH* species over the Ru (001) and $Zr_3O_6H_5$/Ru (001) surfaces with respect to gas phase H_2O and H_2.

Site	$E_{ads, OH}$/eV
The Ru site of Ru (001)	-0.26
The Zr site of $Zr_3O_6H_5$/Ru (001)	-1.33

Supplementary References

1. Yu, H. *et al.* Direct production of olefins from syngas with ultrahigh carbon efficiency. *Nat. Commun.* **13**, 5987 (2022).
2. Zhang, Y. *et al.* Tuning reactivity of Fischer-Tropsch synthesis by regulating TiO_x overlayer over Ru/TiO_2 nanocatalysts. *Nat. Commun.* **11**, 3185 (2020).
3. Kikuchi, E., Matsumoto, M., Takahashi, T., Machino, A. & Morita, Y. Fischer-tropsch synthesis over titania-supported ruthenium catalysts. *Appl. Catal.* **10**, 251-260 (1984).
4. Lian, C., Yu, Y., Zhang, K., Gao, A. & Wang, Y. Highly efficient Fischer–Tropsch synthesis over an alumina-supported ruthenium catalyst. *Catal. Sci. Technol.* **8**, 1528-1534 (2018).
5. González-Carballo, J. M. *et al.* In-situ study of the promotional effect of chlorine on the Fischer–Tropsch synthesis with Ru/Al_2O_3. *J. Catal.* **332**, 177-186 (2015).
6. Koh, T., Koo, H. M., Yu, T., Lim, B. & Bae, J. W. Roles of ruthenium–support interactions of size-controlled ruthenium nanoparticles for the product distribution of Fischer–Tropsch synthesis. *ACS Catal.* **4**, 1054-1060 (2014).
7. Hwang, J. *et al.* Direct confinement of Ru nanoparticles inside nanochannels of large pore mesoporous aluminosilicate for Fischer–Tropsch synthesis. *J. Mater. Chem. A* **3**, 23725-23731 (2015).
8. Kang, J. *et al.* Mesoporous zeolite-supported ruthenium nanoparticles as highly selective Fischer-Tropsch catalysts for the production of C_5-C_{11} isoparaffins. *Angew. Chem. Int. Ed.* **50**, 5200-5203 (2011).
9. Cheng, K. *et al.* Mesoporous beta zeolite-supported ruthenium nanoparticles for selective conversion of synthesis gas to C_5–C_{11} isoparaffins. *ACS Catal.* **2**, 441-449 (2012).
10. Sun, J. *et al.* Highly-dispersed metallic Ru nanoparticles sputtered on H-Beta zeolite for directly converting syngas to middle isoparaffins. *ACS Catal.* **4**, 1-8 (2013).
11. Kang, J., Zhang, S., Zhang, Q. & Wang, Y. Ruthenium nanoparticles supported on carbon nanotubes as efficient catalysts for selective conversion of synthesis gas to diesel fuel. *Angew. Chem. Int. Ed.* **48**, 2565-2568 (2009).
12. Phaahlamohlaka, T. N., Kumi, D. O., Dlamini, M. W., Jewell, L. L. & Coville, N. J. Ruthenium nanoparticles encapsulated inside porous hollow carbon spheres: A novel catalyst for Fischer–Tropsch synthesis. *Catal. Today* **275**, 76-83 (2016).
13. Lyu, S. *et al.* Dopamine sacrificial coating strategy driving formation of highly active surface-exposed Ru sites on Ru/TiO_2 catalysts in Fischer–Tropsch synthesis. *Appl. Catal., B* **278**, 119261(2020).
14. Yang, X. *et al.* Effect of confinement of TiO_2 nanotubes over the Ru nanoparticles on Fischer-Tropsch synthesis. *Appl. Catal., A* **526**, 45-52 (2016).
15. Eslava, J. L., Sun, X., Gascon, J., Kapteijn, F. & Rodríguez-Ramos, I.

Ruthenium particle size and cesium promotion effects in Fischer–Tropsch synthesis over high-surface-area graphite supported catalysts. *Catal. Sci. Technol.* **7**, 1235-1244 (2017).

16. Xiong, K., Li, J., Liew, K. & Zhan, X. Preparation and characterization of stable Ru nanoparticles embedded on the ordered mesoporous carbon material for applications in Fischer–Tropsch synthesis. *Appl. Catal., A* **389**, 173-178 (2010).
